# Respiratory viral and bacterial co-infections in adults in acute care: A multiplex PCR-based study

**DOI:** 10.1371/journal.pone.0358841

**Published:** 2026-09-25

**Authors:** Elina Saarela, Marjo Renko, Matti Uhari, Tytti Pokka, Heikki Kauma, Terhi Ruuska-Loewald

**Affiliations:** 1 Research Unit of Biomedicine and Internal Medicine, University of Oulu and Medical Research Center Oulu, Oulu University Hospital, Oulu, Finland; 2 Department of Pediatrics, University of Eastern Finland and Kuopio University Hospital, Kuopio, Finland; 3 Research Unit of Clinical Medicine, University of Oulu, Oulu, Finland; 4 Research Service Unit, Oulu University Hospital, Oulu, Finland; 5 Department of Pediatrics and Adolescent Medicine, Oulu University Hospital, Oulu, Finland; Debre Markos University, ETHIOPIA

## Abstract

**Background:**

Data on the clinical significance of viral-bacterial co-detection by molecular methods in adults is limited. We aimed to determine how often respiratory viruses and bacteria are co-detected in acutely ill adults and whether co-detection is associated with worse clinical outcomes than detection of a virus or bacterium alone.

**Method:**

We prospectively enrolled 998 adult patients presenting to the emergency department with symptoms of respiratory infection, fever, chest pain, or poor general condition. Nasal swab samples were taken at entry and analysed by multiplex PCR for 16 respiratory viruses. PCR for 7 bacterial pathogens was performed later on 912 frozen-thawed samples. Medical records were reviewed for the clinical outcomes. Analysis of variance (ANOVA) was used to compare the differences in CRP and age between detection groups. The risk of major outcomes was evaluated using multivariate logistic regression.

**Results:**

Both viral and bacterial PCR were positive in 36 of 912 patients (4%). The most common virus in the Co-detection group was rhinovirus (21/36, 58%) and the most common bacterium *Streptococcus pneumoniae* (20/36, 56%)*.* The patients in the Co-detection group were younger than those in the Sole bacteria group, who in turn were younger than those in the Sole virus group. Relative to the Negative group, the risk of pneumonia was highest in the Sole bacteria group (aOR 3.53, p < 0.01) and lower, though still elevated, in the Co-detection group (aOR 2.36, p = 0.03). The risk for antibiotic treatment during 30-day follow-up was greatest in the Co-detection group (aOR 4.42), followed by the Sole bacteria (aOR 2.40) and Sole virus (aOR 2.15) groups (all p < 0.01). There was no significant difference between the groups in the risk of death or ICU care.

**Conclusion:**

Viral and bacterial co-detection was rare in acutely ill adults. Although co-detection was associated with the highest risk of antibiotic treatment, it did not carry the highest risk of pneumonia and was not associated with death or ICU admission. Routine use of multiplex PCR for detecting respiratory viral and bacterial pathogens is therefore unlikely to add clinical value in this population. Larger cohorts powered for severe outcomes and real-time result reporting, are needed to determine whether respiratory viral bacterial testing can meaningfully guide clinical management.

## Introduction

Severe respiratory infections frequently involve more than one pathogen. Analyses of fatalities from the 1918–1919 influenza pandemic demonstrated that secondary bacterial infection, rather than virus alone, accounted for much of the mortality [1]. The clinical relevance of viral-bacterial co-infection has since been confirmed in later pandemics. During the 2009 influenza A (H1N1) pandemic, critically ill patients with bacterial co-infection had higher hospital mortality than those without (31% vs. 21%) [2]. In a systematic review the reported occurrence of bacterial co-infection in influenza patients has ranged from 2% in newborn infants up to 65% in hospitalized adults [3]. Beyond influenza, viral-bacterial interactions has been studied mainly in patients with community acquired pneumonia (CAP) [4–7], where respiratory viruses are detected in 25–29% of patients, and mixed viral-bacterial infection is associated with worse outcomes. A meta-analysis reported an odds ratio for death of 2.1 (95% CI 1.32–3.31) compared with single-pathogen infection [7]. The occurrence and spectrum of detected pathogens also vary with age, with the highest proportion of viral-bacterial detections reported in children and older adults [8]. Several mechanisms may explain these poorer outcomes, including disruption of physical barriers, ciliary dysfunction, enhanced adherence of bacterial pathogens to virus-infected cells, and dysregulation of immune responses [9,10].

Most of the earlier evidence concerning viral-bacterial co-infections predates molecular diagnostics and relied on viral antigen detection and bacterial culture [11,12]. More recently, in a large cohort of hospitalized adults tested with PCR for four respiratory viruses together with bacterial cultures, viral-bacterial co-infection was associated with higher mortality and morbidity than viral or bacterial infection alone [13]. Multiplex polymerase chain reaction (PCR) now allows simultaneous detection of multiple respiratory viruses and bacteria with greater sensitivity and simpler sampling. The enhanced sensitivity, however, may blur the line between clinically meaningful co-infection and incidental co-detection of microbial nucleic acid. A recent large retrospective laboratory cohort using multiplex PCR for both viral and bacterial pathogens reported any co-infection in 7.3% of samples, with a higher occurrence in children [14].

We aimed to determine whether multiplex PCR for respiratory viruses and bacteria improves the understanding of co-infections in a prospectively collected cohort of acutely ill adults [15]. We studied the occurrence of dual detections by multiplex PCR and analysed their associations with clinical outcomes.

## Methods

### Study design and setting

**Fig 1:**
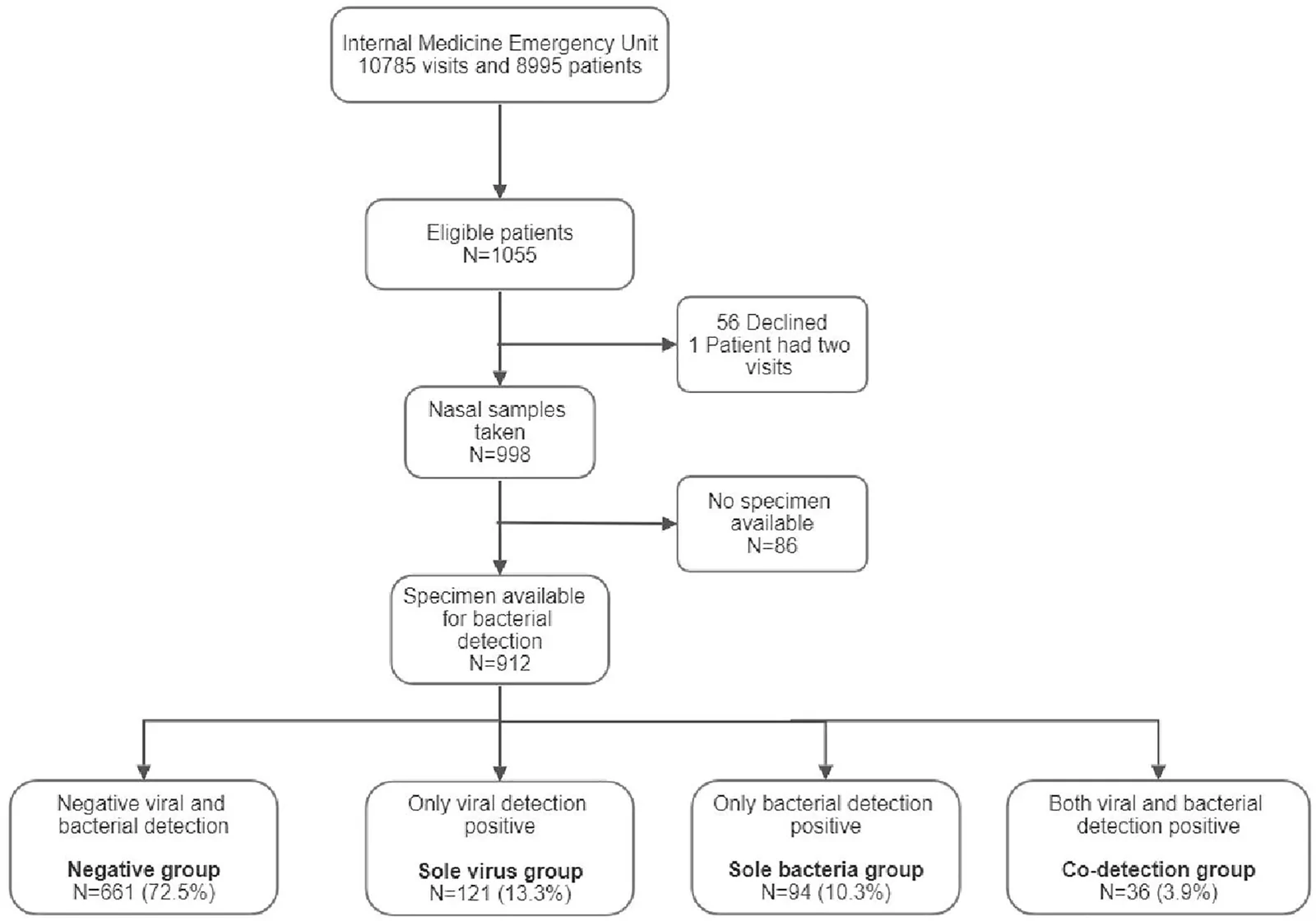
Flow chart of the study and composition of the study groups.

The cohort was collected in connection with a randomized controlled trial concerned with the impact of viral diagnostics in adults [15]. Between September 1, 2014, and September 30, 2015, we enrolled 998 patients who were aged over 16 years, had either symptom of respiratory infection, or a fever (at least 38°C), chest pain or poor general condition for unknown reasons, at the Internal Medicine Emergency Department of Oulu University Hospital (Fig 1). There were no other exclusion criteria except we did not enrol patients who declined to participate. Nasal swab samples were taken by rubbing the nasal mucosa with a dacron stick at a depth of 3–4 centimetres from the eligible patients who had given their written informed consent. Swabs were inserted into a 3 mL Universal Transport Medium tube (Copan Diagnostics, Inc., CA, USA) and sent to the clinical microbiology laboratory at Oulu University Hospital (Nordlab, Oulu, Finland). These samples were then analysed within 72 hours for 16 respiratory viruses with the Anyplex ^TM^ II RV16 Detection kit (Seegene, Seoul, Korea) (Table 1). After this the samples were frozen at −70°C. In 2018–2019 the stored samples were thawed and analysed with the Allplex ^TM^ Respiratory Panel 4 (Seegene, Seoul, Korea) for seven bacterial respiratory pathogens (Table 1). DNA of clinical samples has shown to remain stable even during extended storage [16]. In our own studies nasopharyngeal samples from acutely ill children (n = 1195) and adults (n = 912) were both obtained and analysed at the same time for respiratory bacteria. The proportion of study subjects with a positive bacterial detection was 50% in children and 14% in adults [17,18]. The data was first accessed for research purposes October 1, 2014, and the last access was on January 24, 2019, when the results of bacterial detections were available for the study physicians. Analyses of the data were completed in 2019–2023.

**Table 1:** Coverage of the respiratory pathogen panels used in the study.

Viruses	Bacteria
Adenovirus	*Haemophilus influenzae*
Bocavirus	*Streptococcus pneumoniae*
Coronaviruses OC4/NL63/229E3	*Chlamydia pneumoniae*
Enterovirus	*Mycoplasma pneumoniae*
Influenza A/B viruses	*Legionella pneumophila*
Metapneumovirus	*Bordetella pertussis*
Parainfluenza viruses 1/2/3/4	*Bordetella parapertussis*
Respiratory syncytial viruses A/B	
Rhinovirus	

### Ethical consideration

This study was performed in line with the principles of the declaration of Helsinki. The Ethics Committee of the Northern Ostrobothnia Hospital District, Oulu, Finland, has evaluated the protocol and found it ethically acceptable (decision number EETTMK 45/2014 §137).

### Clinical data collection

At the time of enrolment, study nurse together with the physician working on this research project collected data on comorbidities, current medication and symptoms of the current illness. Another inquiry was done two weeks later focusing on alleviation of the symptoms and the prescription of antibiotics. The study physician also reviewed the medical records. Data on the course of the disease, the length of hospitalization and the duration of intensive care were collected, together with the results of any microbiological tests and radiological examinations. The antimicrobial medication was reviewed from hospital records and from the national electronic database. Together with the participants’ own reports we had the exact duration of 97% of the antibiotic courses. For the episodes with unconfirmed duration, we used the estimate of 10 days. The number of deceased patients was confirmed from the national population register (Statistics Finland). Each patient’s C-reactive protein (CRP) level was analysed as an indicator of inflammation and tissue damage. During data collection authors had access to information that identified individual participants. Since the number of missing data was low we decided not to use any replacement nor imputation.

### Statistical analysis

The sample size of the study was based on the earlier randomized controlled trial [16]. Differences in age at admission between virus-negative and virus-positive patients, and between bacteria-negative and bacteria-positive patients, were tested using Student’s t-test. Analysis of variance (ANOVA) with Tukey’s HSD correction for post hoc tests was used to compare differences in CRP and age between the detection groups (Sole virus, Sole bacteria, Co-detection, Negative). The occurrence of symptoms between detection groups was tested using the chi-squared test. Age and sex adjusted multivariate logistic regression was used to evaluate the risk of major outcomes in the Sole virus, Sole bacteria and Co-detection groups with the Negative group as a reference. Due to our low number in the final analyses it is not possible to adjust more than two variables (age, sex) in the logistic regression analyses. Statistical analysis tool used for the analysis was IBM SPSS Statistics for Windows, version 29 (IBM Corp., Armonk, NY, USA). The figures were drawn using OriginPro 2020 software (OriginLab Corporation, Massachusetts, USA), and the flow chart was drawn online using SmartDraw software (SmartDraw software, LLC, Texas, USA).

## Results

### Microbial findings

The cohort of 912 patients included 661 (72%) who had neither a virus nor bacteria detected in their nasal swab sample ((Negative group) Fig 1). By contrast, a virus was detected in 121 (13%) cases (Sole virus group) and bacteria in 94 (10%) cases (Sole bacteria group). Both the viral and the bacterial PCR were positive in 36 (4%) patients ((Co-detection group) Fig 1).

**Fig 2:**
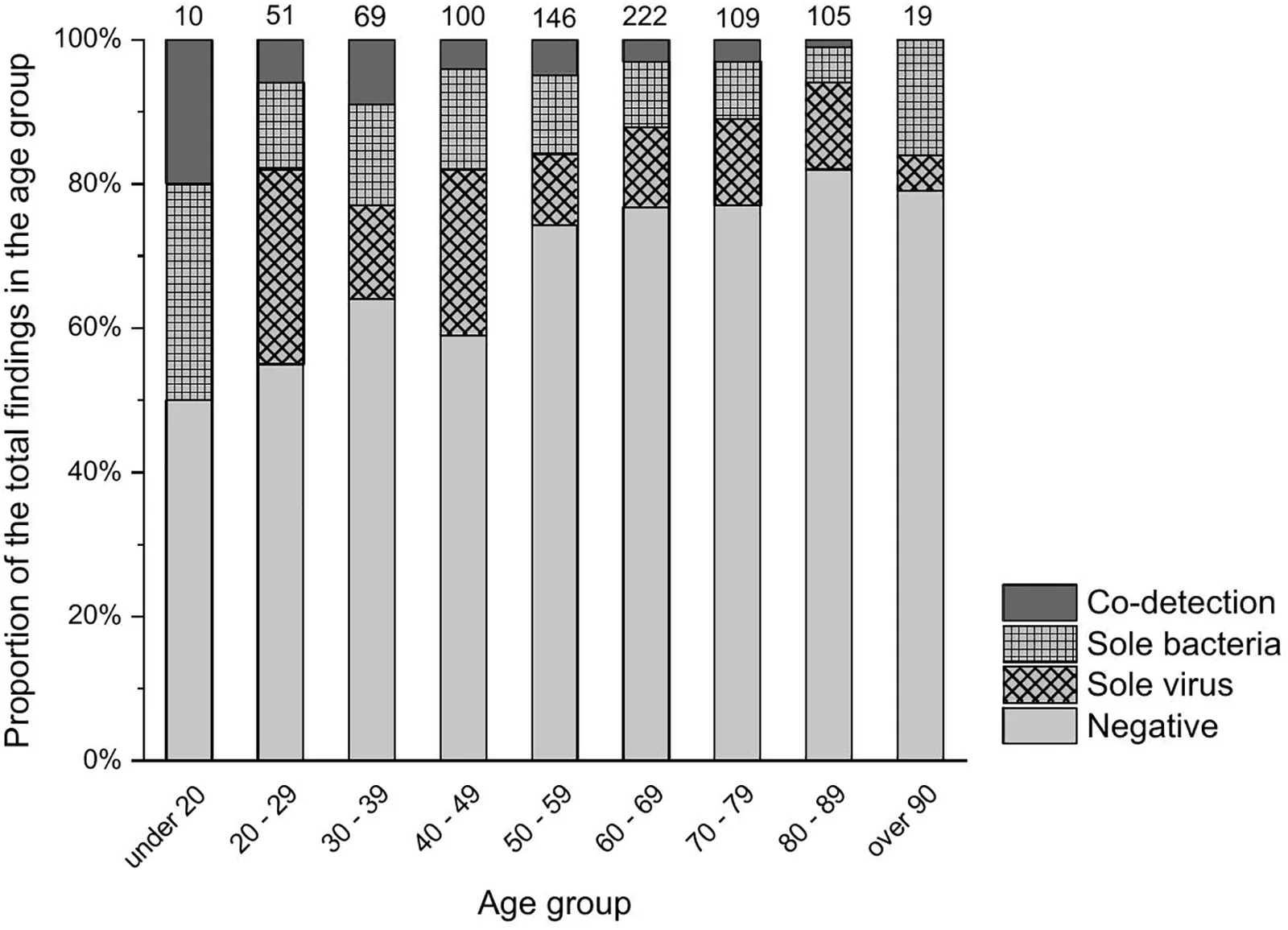
Proportions of viral and bacterial detections by age group. The distributions of patients with given PCR detection results are shown by age group. Each column represents one age group and the detection results are indicated with lighter grey for the Negative group, oblique lines for the Sole virus group, crisscross shading for the Sole bacteria group and darker grey for the Co-detection group. The total number of patients in each age group is marked above the column.

Of all patients, 72% (657/912) had a comorbid illness, most often a chronic lung or heart disease (Table 2). Altogether 611/912 patients (67%) were admitted to hospital, including 454/661 (69%) in the Negative group, 70/121 (58%) in the Sole virus group, 64/94 (68%) in the Sole bacteria group and 23/36 (64%) in the Co-detection group. The mean age was highest in the Negative group, 63.1 (SD 16.7) years, while the patients in the Sole virus group were younger (mean age 57.5 years, SD 18.7, difference 5.5 years, CI 95% 0.8–10.2, P = 0.02), as were those in the Sole bacteria group (mean age 56.4 years, SD 18.3, difference 6.6 years, CI 95% 1.4–11.8, P = 0.01). Those in the Co-detection group were the youngest of all, with a mean age of 52.6 years (SD 18.7, difference 10.5 years, CI 95% 2.0–19.0, P = 0.01). The proportion of negative detection results was higher in the older age groups (Fig 2). Only two patients, both in the Negative group, had received antivirals before study entry. Together 18 patients received oseltamivir during the study period, 1 in the Negative group, 12 in the Sole virus group, 3 in the Sole Bacteria, and 2 in the Co-detection group.

**Table 2:** Background characteristics by PCR detection group.

	All N = 912	Negative group N = 661	Sole virus N = 121	Sole bacteria N = 94	Co-detection N = 36
Age (years), mean (SD)	61.2 (17.5)	63.1 (16.7)	57.5 (18.7)	56.4 (18.3)	52.6 (18.7)
Male	481 (52.7%)	360 (54.5%)	55 (45.5%)	51 (54.3%)	15 (41.7%)
Any comorbidity^a^	657 (72.0%)	486 (73.5%)	81 (66.9%)	64 (68.1%)	26 (72.2%)
CRP at entry (mg/l), mean (SD)	66 (81)	59 (78)	58 (67)	109 (95)	112 (88)
Thoracic imaging performed^b^	856 (93.9%)	619 (93.6%)	113 (93.4%)	88 (93.6%)	36 (100%)
Pneumonia^c^	147 (16.1%)	88 (13.3%)	14 (11.6%)	34 (36.2%)	11 (30.6%)
Antibiotic treatment^d^	566 (62.1%)	373 (56.4%)	90 (74.4%)	72 (76.6%)	31 (86.1%)
Admitted to hospital	611 (67.0%)	454 (68.7%)	70 (57.9%)	64 (68.1%)	23 (63.9%)
Respiratory support^e^	309 (33.9%)	230 (34.8%)	43 (35.5%)	24 (25.5%)	12 (33.3%)
ICU care	55 (6.0%)	44 (6.7%)	7 (5.8%)	3 (3.2%)	1 (2.8%)
Death	29 (3.2%)	25 (3.8%)	2 (1.7%)	2 (2.1%)	0

Abbreviations: CRP C-reactive protein, ICU intensive care unit.

^a^Including chronic lung disease, chronic heart disease, chronic neurological disease, immunosuppression and diabetes.

^b^Either chest X ray or thoracic CT scan.

^c^Pneumonia defined as radiologicist’s interpretation from either chest X-ray or thoracic CT.

^d^Antibiotic treatment during the 30 day follow up.

^e^Includes supportive oxygen, non-invasive ventilation and ventilator treatment.

### Respiratory pathogen findings in the Co-detection group

The most common virus identified in the 36 patients who formed the viral-bacterial Co-detection group was rhinovirus, which was present in 21 samples and the most common bacterium was *S. pneumoniae* with 20 positive detections (Table 3). The combinations of rhinovirus and *S. pneumoniae* and rhinovirus and *H. influenzae* were both found in eight samples. Three patients had three pathogens each (Table 3), comprising one with two viruses (rhinovirus and coronavirus) and *S. pneumoniae*, one with rhinovirus, *S. pneumoniae* and *H. influenzae* and one with coronavirus, *S. pneumoniae* and *H. influenzae*.

**Table 3:** Viral and bacterial results of the 36 patients with both virus and bacteria in their sample.

	Streptococcus pneumoniae* n = 20	Haemophilus influenzae* n = 15	*Chlamydia pneumoniae* n = 3
Rhinovirus*(n = 21)	8	8	3
Metapneumovirus (n = 7)	5	2	0
Coronavirus* (n = 5)	2	1	0
Influenzavirus (n = 4)	2	2	0

Patients (n = 33) with two pathogens detected in their nasal samples are shown in the table. Additional three patients had three different pathogens detected in their samples. The numbers depicted below the pathogens are the total numbers of positive detections for each pathogen. The triple co-detection pathogens are marked with *.

### Symptoms

At entry, 80% (527/661) of the patients in the Negative group, 94% (114/121) in the Sole virus group and 88% (83/94) in the Sole bacteria group had a cough, rhinitis, a sore throat, or shortness of breath, while all 36 members of the Co-detection group had at least one respiratory symptom. Other symptoms of infection, including fever, chest pain and fatigue or poor general condition, were reported by 96% (875/912) of the participants. There were no clinically significant differences in symptomology between the groups.

### Differences in CRP between the groups

The CRP level at entry was significantly higher in the Sole bacteria group than in the Negative group (109 mg/l vs. 59 mg/l, difference of means 49 mg/l, CI 95% 22 mg/l to 76 mg/l, P < 0.01) and similarly in the Co-detection group compared with the Negative group (112 mg/l vs. 59 mg/l, CI 95% 13 mg/l to 93 mg/l, P = 0.01). Furthermore, CRP was significantly higher in the Sole bacteria and Co-detection groups than in the Sole virus group (109 mg/l vs. 58 mg/l, difference of means 50 mg/l, CI 95% 20 mg/l to 81 mg/l, P < 0.01 and 112 mg/l vs. 58 mg/l, difference of means 54 mg/l, CI 95% 12 mg/l to 96 mg/l, P = 0.01).

### Clinical outcomes

The risk of pneumonia was 3.5-fold in the Sole bacteria group (CI 95% 2.16–5.76, P < 0.01) relative to the Negative group and 2.4-fold in the Co-detection group (CI 95% 1.11–5.02, P = 0.03, Table 4). The risk of requiring antibiotic treatment during the 30-day follow-up relative to the Negative group was 2.15 (CI 95% 1.39–3.34, P < 0.01) in the Sole virus group, 2.40 (CI 95% 1.45–3.98, P < 0.01) in the Sole bacteria group and 4.42 (CI 95% 1.69–11.55, P < 0.01, Table 4) in the Co-detection group. There was no significant difference between the groups in the risk of either death or requiring ICU care. We tested only clinically relevant outcomes according to our hypothesis; hence we did not use any post test corrections.

**Table 4:** Comparison of major outcomes according to the respiratory pathogens detected with Negative microbial group as the reference level.

	Sole virus group			Sole bacteria group			Co-detection group		
	aOR^a^	CI 95%	p	aOR	CI 95%	p	aOR	CI 95%	p
Pneumonia	0.80	0.43–1.47	0.47	3.53	2.16–5.76	<0.01	2.36	1.11–5.02	0.03
Antibiotic treatment	2.15	1.39–3.34	<0.01	2.40	1.45–3.98	<0.01	4.42	1.69–11.55	<0.01
ICU care	0.95	0.41–2.18	0.95	0.46	0.14–1.53	0.21	0.46	0.06–3.45	0.45
Death ^b^	0.72	0.15–3.56	0.69	1.24	0.23–6.59	0.80	0	0	1.0

^a^Adjusted for age and sex.

^b^Within 30 days follow-up time.

## Discussion

In this cohort of acutely ill adults, the proportion of patients with positive viral and bacterial co-detection was low, only 4%, even though one of the selection criteria was a respiratory symptom. In studies focusing the patients with pneumonia, the proportion of patients with viral-bacterial co-detection has been higher, 25–29% [4,7]. Yet, our finding is in line with a previous cohort study of respiratory infection patients, in which the proportion of laboratory-confirmed viral-bacterial co-infections was 6.8% [14].

The most common virus was rhinovirus, in accordance with the literature [19,20]. In the present cohort there were only four co-detection patients with influenza, evidently on account of a quiet influenza season during the winter of 2014–2015. As expected, *S. pneumoniae* was the most prevalent bacterial pathogen, with *H. influenzae* the second most prevalent [4,21,22]. Our panel did not include *S. aureus*, which has been reported to be a major pathogen, especially in influenza patients [3].

Co-detection has been associated earlier with poorer clinical outcomes in the form of severe pneumonia, longer times in mechanical ventilation and even death [4,5]. Since the number of co-detection patients in this cohort was low, the incidences of both ICU care and death were low in all the groups, so that it is not possible to draw relevant conclusions regarding these outcomes.

Co-detection was associated with higher risk of pneumonia, yet the risk of pneumonia was even higher in the Sole bacteria group. The patients with viral-bacterial co-detection had the highest risk of receiving bacterial antibiotics. Since the bacterial detection results were not available for the clinicians during the study period, treatment was based on clinical evaluation. The co-detection is associated with more severe clinical presentations [7,13]. The relatively low percentage of co-detections in this cohort of acutely ill patients suggests that the routine use of bacterial PCR panel in patients with viral infections does not offer substantial benefit for clinical assessment.

Even though the results were not available to the emergency clinician, the probability of receiving antibiotic treatment was higher in the patients with positive detections of respiratory pathogens. One possible explanation may be the CRP value, which was higher in the patients with positive bacterial detection with or without a virus. The CRP has been adopted in clinical laboratories as a sensitive systemic marker of inflammation and tissue damage and is widely used as such in many European countries [23]. Thus, higher CRP has been associated with bacterial co-infection in influenza-like illness and with greater severity of community-acquired pneumonia [24,25].

Chest radiography is often regarded as the reference standard for the diagnosis of community-acquired pneumonia, even though its reliability is limited because of inter-observer variability and confounding factors [26]. In our cohort 94% of the patients had either a chest X-ray or a thoracic CT scan taken and there was a strong association between positive bacterial detection and radiologically defined pneumonia. We presume that radiological diagnosis also increased the prescription of bacterial antibiotics.

The strength of this study lies in the systematic collection of data on the symptoms and clinical course of the disease in each case and on the prescription of antibiotics in a prospective collected clinical cohort of acutely ill adults. We gathered samples from over 900 patients, using non-invasive nasal swabs, providing a material that is highly representative of patients in emergency units, thus making the results widely generalizable. Most of the patients had either a chest X ray or thoracic CT performed, thus providing comprehensive diagnostic data with respect to pneumonia. Small co-detection sample size limited some subgroups analyses. Nasal swab sampling may be regarded as a limitation for diagnosing lower respiratory tract infections, though it was chosen on account of its applicability to the emergency unit setting, in which lower respiratory samples cannot be taken from all patients. Results obtained by multiplex PCR do not distinguish between colonization and infection, particularly when nasopharyngeal sampling is used. The used multiplex PCR panel did not detect all clinically relevant respiratory bacteria such as *Staphylococcus aureus* and *Moraxella catarrhalis*. We lacked reliable data on influenza vaccinations in this cohort.

In conclusion, viral and bacterial co-detection was rare in acutely ill adults. Although co-detection was associated with the highest risk for antibiotic treatment, it did not carry the highest risk of pneumonia and was not associated with death or ICU admission. Based on the present study, routine use of multiplex PCR for detecting respiratory viral and bacterial pathogens is therefore unlikely to add clinical value in this population. Future studies should include larger, preferably multicentre cohorts powered for severe outcomes such as intensive care and death. Studies in which detection results are available to clinicians in real time would further clarify whether multiplex respiratory PCR adds value in guiding antibiotic treatment.
